# An Integrated Strategy of UHPLC-ESI-MS/MS Combined with Bioactivity-Based Molecular Networking for Identification of Antitumoral Withanolides from *Athenaea fasciculata* (Vell.) I.M.C. Rodrigues & Stehmann

**DOI:** 10.3390/molecules29184357

**Published:** 2024-09-13

**Authors:** André Mesquita Marques, Lavinia de Carvalho Brito, Simony Carvalho Mendonça, Brendo Araujo Gomes, Flávia da Cunha Camillo, Gustavo Werneck de Souza e Silva, André Luiz Franco Sampaio, Suzana Guimarães Leitão, Maria Raquel Figueiredo

**Affiliations:** 1 Department of Natural Products, Pharmaceutical Technology Institute, Farmanguinhos, Fiocruz, Sizenando Nabuco 100 st, Manguinhos, Rio de Janeiro 21041-250, Brazil; andrefarmaciarj@yahoo.com.br (A.M.M.); flavia.camillo@fiocruz.br (F.d.C.C.); maria.figueiredo@fiocruz.br (M.R.F.); 2 Department of Natural Products and Food, Faculty of Pharmacy, Center of Health Sciences (CCS), Federal University of Rio de Janeiro, Rio de Janeiro 21941-902, Brazil; sy2802@gmail.com (S.C.M.); brendoo.bc@gmail.com (B.A.G.); sgleitao@gmail.com (S.G.L.); 3 Laboratory of Molecular Pharmacology, Pharmaceutical Technology Institute, Farmanguinhos, Fiocruz, Avenida Brasil 4365, Manguinhos, Rio de Janeiro 21041-250, Brazil; gustavo.werneck@fiocruz.br (G.W.d.S.e.S.); andre.sampaio@fiocruz.br (A.L.F.S.)

**Keywords:** *Aureliana fasciculata*, GNPS, cancer, Solanaceae, withanolides

## Abstract

Background: *Athenaea fasciculata*, a Brazilian native species from the Solanaceae family, is recognized as a promising source of bioactive withanolides, particularly Aurelianolide A and B, which exhibit significant antitumoral activities. Despite its potential, research on the chemical constituents of this species remains limited. This study aimed to dereplicate extracts and partitions of *A. fasciculata* to streamline the discovery of bioactive withanolides. Methods: Using ultra-high-performance liquid chromatography–tandem mass spectrometry (UHPLC-MS/MS), various extracts—including *n*-hexane, methanol, and ethanol—were analyzed, and their mass spectrometry data were processed through the GNPS platform for the generation of molecular networking. The results indicated that crude extracts displayed comparable cytotoxicity against Jurkat cells, by treatment at 150 µg/mL, while alcoholic extracts achieved approximately 80% inhibition of K562 cells and K562-Lucena 1 at the same concentration. Notably, the dichloromethane partition exhibited the highest cytotoxicity across leukemia cell lines, particularly against Jurkat cells (IC_50_ = 14.34 µg/mL). A total of 22 compounds were annotated by manual inspection and different libraries, with six of them demonstrating significant cytotoxic effects. Conclusions: This research underscores the therapeutic potential of *A. fasciculata* and highlights the effectiveness of integrating advanced analytical methods in drug discovery, paving the way for further exploration of its bioactive compounds.

## 1. Introduction

Natural products have long been acknowledged in pharmaceutical research as a promising reservoir for discovering novel therapeutic agents in the management of diverse diseases, including cancer [1]. Noteworthy examples of cancer drugs include vincristine, podophyllotoxin derivatives, and paclitaxel (Taxol) and ongoing investigations are exploring numerous other natural compounds, thus promoting a continuous exploration of natural products as potential anticancer agents [2].

**Figure 1:**
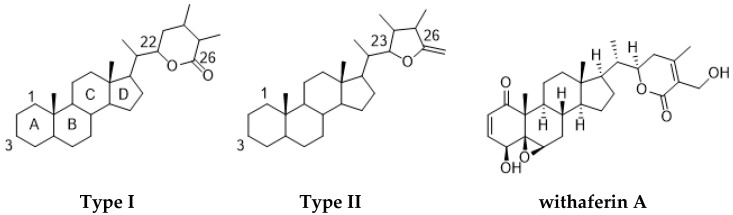
Chemical structures of type I and II withanolides and withaferin A.

Among the natural products, withanolide class compounds stand out as promising anticancer agents, besides having other valuable reported pharmacological properties. Until now, more than 650 withanolides have been described, with most of these from members of the Solanaceae family. Withanolide metabolites are steroidal triterpene lactones composed of an ergostane-type skeleton comprising 28 carbon atoms, with either C-26 and C-22 or C-26 and C-23 oxidized to form a δ-lactone ring (type I) or a γ-lactone ring (type II), respectively. These rings are attached to an intact or rearranged ergostane scaffold. Present in many derivatives, the oxygenation at C-1 generates 1-oxo-vitanolides such as withaferin A, the first withanolide to be discovered (Figure 1) [3,4]. 

**Figure 2:**
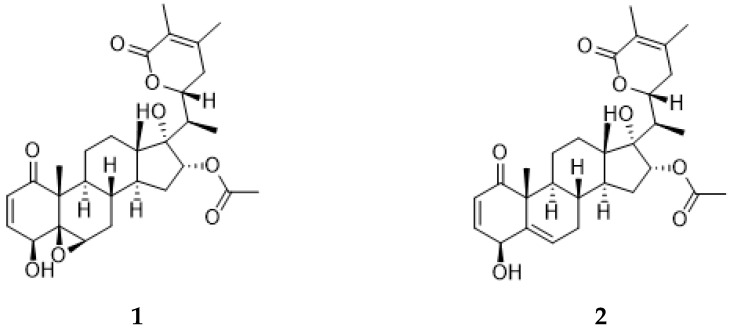
Aurelianolides A (**1**) and B (**2**).

*Athenaea fasciculata*, previously referred to as *Aureliana fasciculata*, is a native Brazilian species that had its name re-established as the correct taxon name to *Athenaea*, belonging to the Solanaceae family [5]. Phytochemical studies on the isolation of secondary metabolites from this species are sparse in the literature; however, two major type I withanolides, aurelianolides A and B (Figure 2), have been isolated from its dichloromethane partition [6].

In the last few years, some studies have assessed the pharmacological potential of withanolide compounds with promising results. Using a panel of leukemia cell lines, it was demonstrated that aurelianolide A displayed interesting cytotoxic activity against MOLT-4 cells (IC_50_ = 1.17 μM), while aurelianolide B showed a stronger effect on Jurkat cells (IC_50_ = 2.25 μM). Caspase activation was suggested as the key to apoptosis induction in Jurkat and K562-Lucena 1 cell lines, without necrosis induction [7]. Besides their anticancer activity, these compounds have also demonstrated significant toxic effects against tropical neglected disease parasites. Their potential leishmanicidal effects against promastigotes of *L. amazonensis* were evaluated as IC_50_ 7.61 μM and 7.94 μM, while against the intracellular amastigotes, they displayed IC_50_ values of 2.25 M and 6.43 M. In accordance with in silico predictions, aurelianolides A and B have good bioavailability via the oral route, showing a similar effect to miltefosine, the reference drug, and presenting low toxicity [8]. In another study, these two compounds showed EC_50_ values of 4.6 ± 1.3 μM and 1.6 ± 0.4 μM, respectively, against intracellular forms of *Trypanosoma cruzi*. Their ADMET parameters in silico revealed comparable results to benznidazole (Bz), the reference drug, while avoiding mutagenicity and hepatotoxicity associated with Bz [9]. 

Over the last decades, different areas of Chemistry and Pharmacology have sought to understand the specific functions of natural products, as well as their potential use as therapeutic agents. Technological advances in analytical platforms combined with increased data processing capacity expanded the view of the metabolic functions of plants and their roles as alternative sources of chemically diverse bioactive metabolites. However, the unambiguous annotation and/or elucidation of “the entire” set of metabolites present in a biological system is still considered a challenging task in analytical terms. Currently, there is no single analytical technique able to measure the wide diversity of metabolites in a single experiment. Thus, orthogonal analytical techniques are needed to overcome individual deficiencies [10]. The application of ultra-high-performance liquid chromatography coupled with electrospray with tandem (UHPLC-ESI-MS/MS) became an effective tool to rapidly characterize plant-extracted chemical constituents [11]. Additionally, the integration of this technique with the Global Natural Product Social (GNPS) platform has streamlined the processing and interpretation of extensive MS2 spectral datasets, particularly in complex matrices of plant extracts and their partitions. This combination allows a comprehensive study of chemical profiles and facilitates bioactivity-based molecular networking, offering a viable and efficient methodology for natural product research [12,13]. Therefore, the present study represents the first systematic investigation of *Athenaea fasciculata* extracts and partitions to determine its phytochemical profile using UHPLC-MS/MS and molecular networking techniques with a focus on correlating the withanolides content with cytotoxicity against leukemia cells.

## 2. Results and Discussion

### 2.1. Cytotoxic Activity of A. fasciculata Extracts and Partitions against Human Leukemia Cell Lines

**Figure 3:**
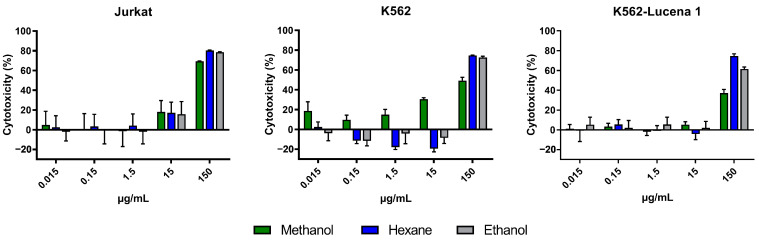
Assessment of in vitro cytotoxicity of *A. fasciculata* crude extracts against Jurkat (**A**), K562 (**B**), and K562-Lucena 1 (**C**) leukemia cell lines using MTT technique as described in the Methods Section. Points represent the mean ± standard error of the mean (SEM) of at least two independent experiments.

**Figure 4:**
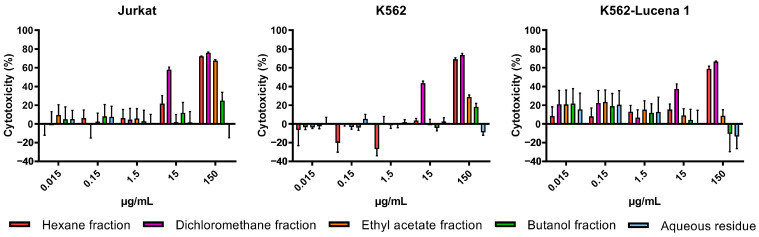
Assessment of in vitro cytotoxicity of *A. fasciculata* ethanolic partitions against Jurkat (**A**), K562 (**B**), and K562-Lucena 1 (**C**) leukemia cell lines using MTT technique as described in the Methods Section. Points represent the mean ± standard error of the mean (SEM) of at least two independent experiments.

The cytotoxicity of crude extracts obtained with *n*-hexane (AFFH), ethanol (AFFE), and methanol (AFFM) (Figure 3), and ethanol extract partitions (AFFPH, AFFD, AFFAc, AFFBu, and AFFAq) (Figure 4) was evaluated against Jurkat, K562, and K562-Lucena 1 human leukemia cell lines at concentrations ranging from 0.015 µg/mL to 150 µg/mL.

*A. fasciculata* crude extracts exerted cytotoxicity against Jurkat and K562 cells, and on the multidrug-resistant (MDR) cell line K562-Lucena 1. We can observe that at 150 µg/mL, all extracts have a similar cytotoxic effect on the Jurkat cell line (Figure 3A). On the K562 cell line, methanolic and ethanolic extracts reached ~80% inhibition at 150 µg/mL; a similar performance was seen on the MDR cell line K562-Lucena 1 (Figure 3B,C). After calculation of the IC_50_ for the crude extracts, it was observed that the Jurkat cell line was more susceptible to treatment with *A. fasciculata* crude extracts, with the methanolic extract being less active in general, with a higher IC_50_ (Table 1). Despite similar IC_50_ values for *n*-hexane and ethanolic extracts, we used the ethanolic extract partition for further analysis of cytotoxic effect.

Cells were treated with *A. fasciculata* extracts and partitions (from 0.015 µg/mL to 150 µg/mL) for cytotoxicity assessment using MTT as described in the Section 3.3. IC_50_ calculations were performed using GraphPad Prism ver. 5 (San Diego, CA, USA).

Among ethanolic extract partitions, the AFFD partition demonstrated the highest cytotoxicity, evidenced by the lower IC_50_ values across the three leukemia cell lines, with higher activity against Jurkat cells (IC_50_ =14.34 µg/mL). However, AFFAc and AFFBu partitions exhibited comparatively lower cytotoxicity, while the AFFAq, with IC_50_ > 1000 µg/mL, had no relevant activity against all tested cell lines (Table 1; Figure 4). 

Our findings indicate that the AFFD partition is the most relevant bioactive sample, suggesting the presence of antitumoral compounds. In fact, in our previous study, two compounds named Aurelianolides A and B were isolated and characterized as major constituents of the AFFD partition and showed relevant anticancer activity against leukemic cells, mainly Jurkat, K-562, and K-562 Lucena cells, promoting caspase 3/7 activation, and apoptosis in Jurkat and K-562 Lucena 1 cells [7]. In the current study, the nonpolar AFFPH partition showed some cytotoxic activity, which can be attributed to the presence of other relevant active compounds; on the other hand, the AFFAc, AFFBu, and AFFAq partitions, mainly composed of phenolic-derivative compounds such as flavonoids and phenolic acids, had lower or no effects on the human leukemic cells tested.

### 2.2. Ultra-High-Performance Liquid Chromatography–Tandem Mass Spectrometry Analysis 

**Figure 5:**
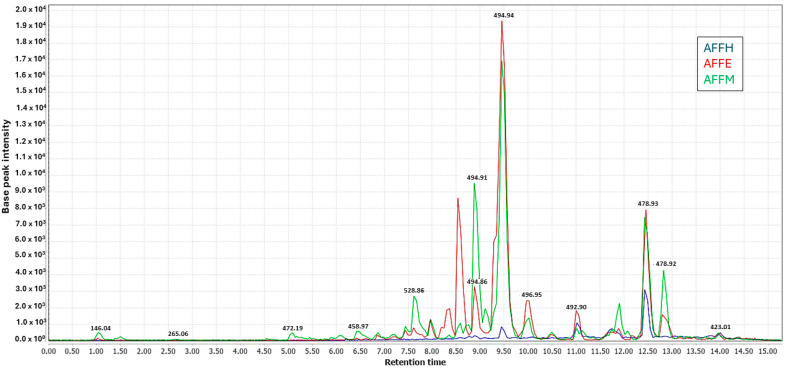
UHPLC-ESI-MS/MS showing overlaid chromatograms of base peaks recorded (Rt 1.0 to 15.0 min) for methanol (AFFM), ethanol (AFFE), and *n*-hexane (AFFH) crude extracts in negative ionization mode.

Three extracts of *A. fasciculata*, along with four partitions from its ethanolic extract and final aqueous residue, were each analyzed in triplicate (24 samples in total). Analysis was conducted using ultra-liquid chromatography coupled with tandem mass spectrometry (LC-MS/MS) with electrospray ionization (ESI) as the ionization source, operating in both positive and negative ionization modes. As expected, the methanolic and ethanolic extracts exhibited better ionization compared to the hexane extract, both in the negative mode (Figure 5). In both ionization modes, the base peak chromatograms of the methanolic and ethanolic extracts shared a similar profile, with some variations observed in peak intensities. 

**Figure 6:**
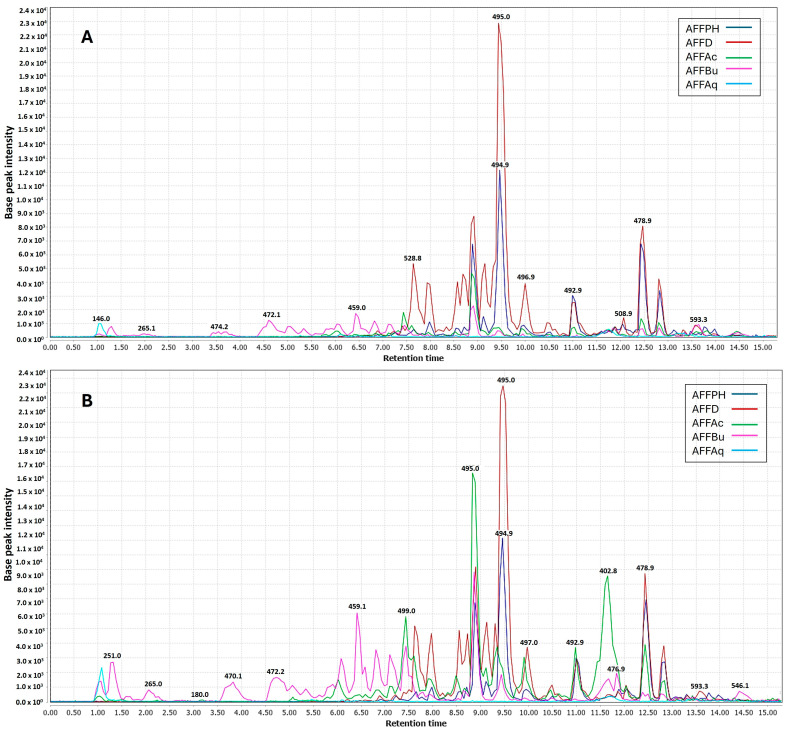
UHPLC-ESI-MS/MS showing overlaid chromatograms of base peaks recorded (Rt 1.0 to 15.0 min) for ethanolic partitions (hexane (AFFPH), dichloromethane (AFFD), ethyl acetate (AFFAc), butanol (AFFBu) fractions, and (AFFAq) aqueous residue in two ionization modes. (**A**) Negative mode. (**B**) Positive mode.

In the context of partitions, negative ionization was more efficient for dichloromethane (AFFD), *n*-hexane (AFFPH), and butanol (AFFBu) partitions (Figure 6A). Positive ionization yielded better results for all partitions, except aqueous residue. Ethyl acetate (AFFAc) and butanol (AFFBu) partition samples were enhanced in the negative mode (Figure 6B).

### 2.3. Chemometric Analyses Using PLS Regression Model

A Partial Least Squares (PLS) regression analysis model was constructed to correlate the LC-MS data with the cytotoxic activity of the extracts and partitions of *A. fasciculate*. The PLS regression utilized the LC-MS dataset (*m*/*z* ions and Retention Time—RT) of 24 samples as independent variables (X), while the dependent variable (Y) reflected cytotoxic activities against the three leukemic cell lines. This algorithm integrates principal component analysis (PCA) and linear regression to identify latent variables optimizing the correlation between predictors and biological activity.

For the independent variables (X) dataset, a low-level data fusion strategy was employed. This involved processing spectral data separately from mass spectrometry analysis using ESI sources in positive and negative ionization modes, normalizing them, concatenating, and forming an independent data matrix. The resulting low-level data matrix, comprising 24 rows (samples) and 211 columns (*m*/*z* ions and Retention Time), including 114 (M + H)^+^ and 97 (M − H)^−^, underwent autoscaling. The dependent variables (Y) dataset comprised one column representing the potential multitarget activity of the samples. The samples were considered multitarget cytotoxic if they exhibited cytotoxic potential (IC_50_ ≤ 100) against at least two investigated leukemia cell lines. Conversely, if IC_50_ ≤ 100 was determined against only one target or against none of the targets, the samples were classified as non-multitarget cytotoxic. Multitarget cytotoxic extracts or partitions were designated as [1], while non-multitarget extracts were denoted as [−1].

**Figure 7:**
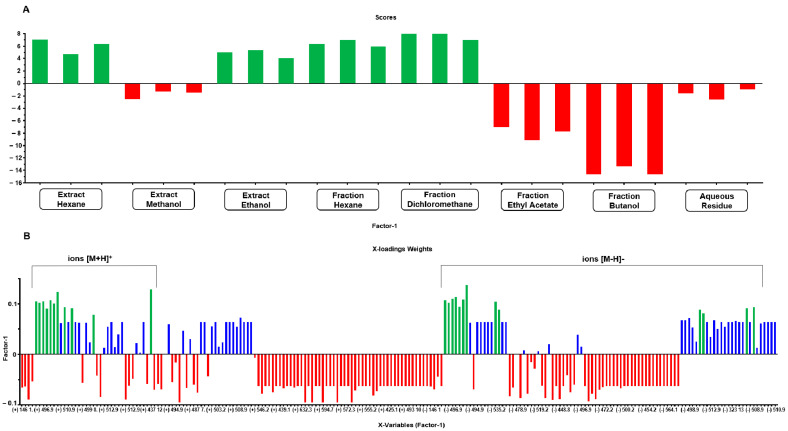
Scores (**A**) and X-loadings weights (**B**) for the PLS model based on the LC-MS data obtained from the low-level data matrix (concatenated ESI [+/−]) of the 24 samples from extracts and partitions of *A. fasciculata*. The most abundant ions were marked with green color, in both positive and negative mode.

The multitarget PLS model (Figure 7) achieved an explained variance of 88.5% with two factors and 99.6% with all factors (seven). The model demonstrated an R^2^ of 0.97 and a Q^2^ of 0.93, indicating a reliable fit; furthermore, it exhibited low RMSE values, with the RMSECV (validation error) at 0.28, slightly higher than the RMSEC (calibration error) at 0.17.

In the PLS model score plot (Figure 7A), the distinction between the two distinct groups (cytotoxic and non-cytotoxic multitarget) is clearly visible. The X-loadings weights plot (Figure 7B) highlights the ions that contribute most positively to explaining the desired activity, specifically those that are either exclusively present or more abundant in extracts with higher activity. This analysis identified 13 ions in negative ionization mode with values [M − H]^−^ *m*/*z* 419.0. 434.9, 478.9, 486.9, 496.9, 508.9, 510.9, 512.8, 519.2, 526.9, 528.9, 542.9, and 560.9, and 11 ions in positive ionization mode with values [M + H]^+^ *m*/*z* 492.9, 494.9, 496.9, 508.9, 510.9, 512.9, 519.2, 526.9, 542.9, 560.9, and 593.3.

### 2.4. Annotated Compounds for the Predicted Ions in the Multitarget Model Using Molecular Networking Analyses

Molecular networking facilitates the analysis of mass spectrometry data by employing algorithms to systematically organize compounds into clusters within the same class based on their mass fragmentation similarities. Following this, dereplication is executed by comparing them with public databases or in-house libraries to annotate known metabolites [14]. GNPS is an open-access web platform for constructing molecular networks from raw mass spectrometry data. It facilitates the organization, processing, and annotation of fragments. Additionally, both raw and processed data can be stored and shared [15,16]. 

Based on this procedure, the 13 ions predicted in the negative ionization mode and the 11 ions predicted in the positive ionization mode were annotated as shown in Table 2 and Table 3. However, two of them were annotated in both positive and negative modes, namely *m*/*z* 508.9 [M − H]^−^ and 510.9 [M + H]^+^, corresponding to compound **8** (physaminilide H), and *m*/*z* 510.9 [M − H]^−^ and 512.9 [M + H]^+^, corresponding to compound **9** (aurelianolide B).

**Table 2:** UHPLC-ESI-MS/MS data for annotated and proposed compounds (undescribed) in the molecular network in negative ion mode (MS/MS spectra in Supplementary Figures S1–S21).

Compound	Rt (min)	Molecular Formula	[M − H]^−^ (*m*/*z*)	MS/MS (MS^2^)	Proposed/Annotated Compound	Reference
**1**	7.2	C_30_H_42_O_8_	528.9	511, 493, 469, 451, 433	virginol A	[17]
**2**	7.4	C_28_H_40_O_7_	486.9	469, 451, 453	16-deoxyphiladelphicalactone C	[18]
**3**	8.7	C_30_H_40_O_9_	542.9	525, 510, 483, 465	withaneomexolide A	[19]
**4**	8.9	C_31_H_46_O_9_	560.9	542, 529, 501, 489	(20S,22R,24S,25S,26R/S)-15α-acetoxy-5,6β:22,26:diepoxy-24-methoxy-4β,25,26-trihydroxyergost-2-en-1-one	[20]
**5**	9.1	C_28_H_36_O_4_	434.9	417, 399, 391, 377	(4S,20S,22R)-4-Hydroxy-1-oxo-witha-2,5,16,24-tetraenolide	[21]
**6**	9.4	C_30_H_42_O_7_	512.8	495, 435, 417	physapubescin H	[22]
**7**	10.6	C_30_H_40_O_8_	526.9	509, 467, 449, 431, 421	aurelianolide A	[6]
**8**	11.2	C_30_H_38_O_7_	508.9	449, 431, 413, 385	physaminilide H	[23]
**9**	11.5	C_30_H_40_O_7_	510.9	492, 475, 463, 451	aurelianolide B	[6]
**10**	12.4	C_28_H_32_O_7_	478.9	461, 419, 401	nicanlode C	[24]
**11**	12.4	C_28_H_36_O_3_	419.0	401, 383, 373	phenowithanolide	[25]
**12**	12.4	C_28_H_40_O_9_	519.2	501, 459, 441	withaperuvin N	[26]
**13**	12.5	C_30_H_42_O_6_	496.9	479, 419	baimantuoluoline R	[27]

**Table 3:** UHPLC-ESI-MS/MS data for annotated and proposed compounds (undescribed) in the molecular network in positive ion mode (MS/MS spectra in Supplementary Figures S1–S21).

Compound	Rt (min)	Molecular Formula	[M + H]^+^ (*m*/*z*)	MS/MS (MS^2^)	Proposed/Annotated Compound	Reference
**8**	7.8	C_30_H_38_O_7_	510.9	493, 451, 433, 340	physaminilide H	[23]
**9**	7.9	C_30_H_40_O_7_	512.9	495, 453, 435, 417	aurelianolide B	[6]
**14**	8.5	C_30_H_38_O_8_	526.9	509, 467, 449, 431	physaminilide B	[28]
**15**	8.7	C_31_H_44_O_9_	560.9	543, 528, 517	physanicandrolide B	[29]
**16**	8.7	C_30_H_38_O_9_	542.9	525, 510, 482, 464	physagulide C	[30]
**17**	9.4	-	494.9	477, 453, 435, 417	not identified	
**18**	11.0	C_28_H_44_O_7_	492.9	475, 433, 415	capsisteroid G	[31]
**19**	11.1	C_28_H_44_O_8_	508.9	491, 473, 449	capsisteroid D	[32]
**20**	12.4	-	496.9	479, 437, 419, 392	not identified	
**21**	12.8	C_29_H_42_O_8_	519.2	501, 459, 441	withalongolide D	[33]
**22**	13.6	-	593.3	575, 565, 533, 505, 461, 433	not identified	

**Figure 8:**
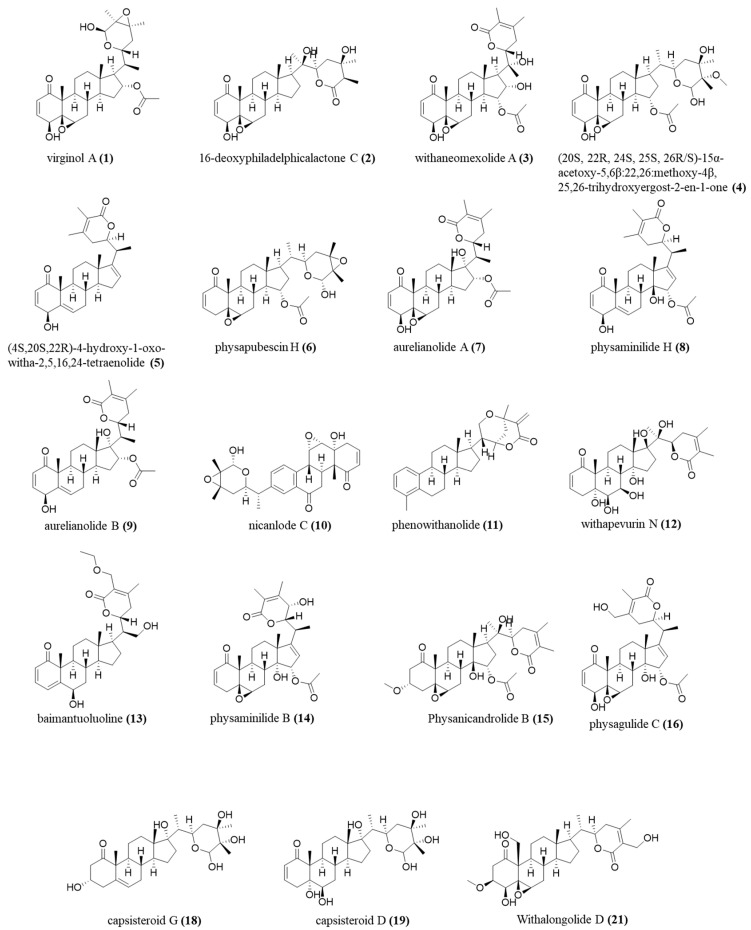
Chemical structure, exact mass, and molecular formula of annotated compounds.

The annotations were made based on the compounds’ fragmentation profile and comparison with the literature; all belong to the class of withanolides, widely found in the studied species (Figure 8). 

**Scheme 1:**
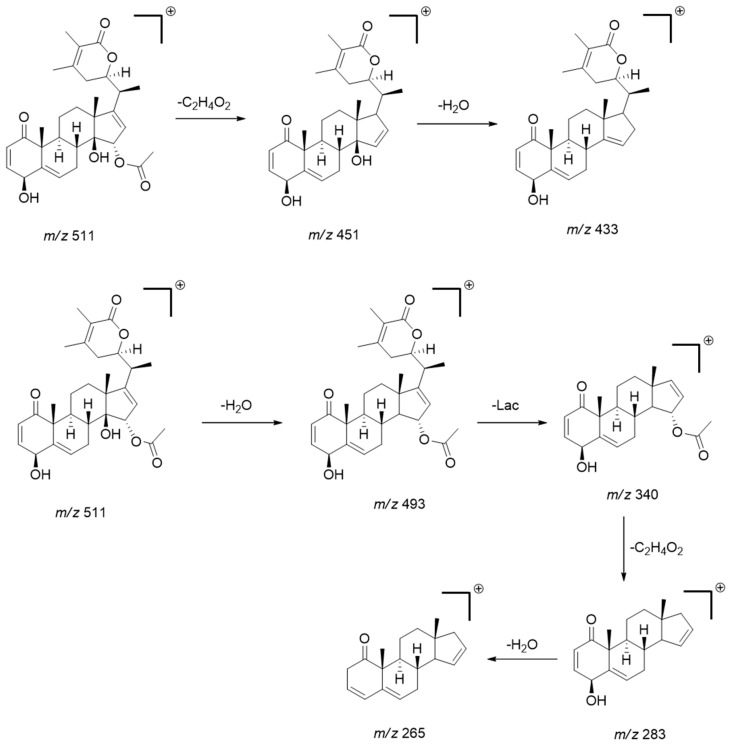
Scheme showing the main fragmentation pattern of withanolides using physaminilide H (compound **8**) as an example. (Lac = lactone fraction) [34].

The MS/MS pattern reveals consecutive losses of H_2_O (−18 Da), acetyl (C_2_H_4_O_2_; −60 Da) or (C_2_H_2_O, −42 Da), methyl (CH_3_, −15 Da), and CO (−28 Da), characteristic of this compound class [34,35]. The diagnostic product ions obtained from the precursors [M + H]^+^ or [M − H]^−^ were mainly generated by the loss of water, acetyl, and subsequent cleavage/rearrangement of the lactone fraction (Lac), as exemplified below for physaminilide H (**8**) (Scheme 1).

**Figure 9:**
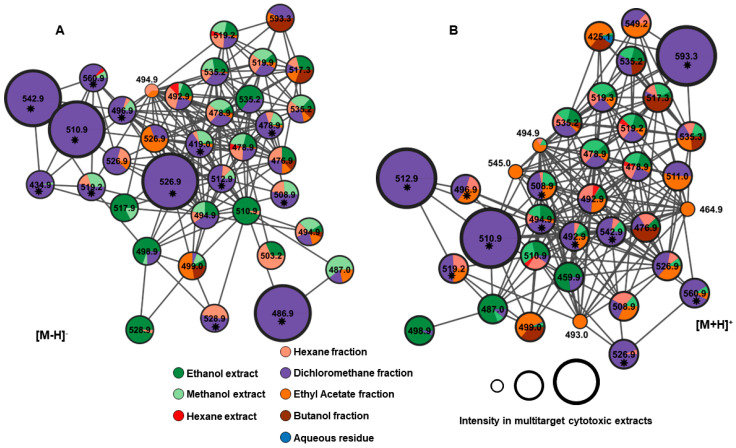
Networks A and B from negative and positive ionization modes, respectively, showing the class of withanolide compounds. * Predicted ions in the PLS model (with the greatest contribution to explaining multitarget cytotoxic activity).

All predicted ions, both in positive and negative modes, were observed in networks A and B as depicted in Figure 9. Furthermore, we can presume that the unannotated compounds might belong to the same class of compounds, as they are grouped based on similarity in the fragmentation profile. The GNPS platform enables the creation of molecular networks that can be used to group compounds into molecular families based on similarities identified through the analysis of MS/MS spectra [16,36].

Among the 24 ions predicted in the PLS model, six compounds contribute most significantly to cytotoxic activity: 16-deoxyphiladelphicalactone C (**2**), withaneomexolide A (**3**), aurelianolide A (**7**), Physaminilide H (**8**), aurelianolide B (**9**), and one unidentified compound (**22**). The corresponding negative and positive ions are depicted in Figure 9.

**Figure 10:**
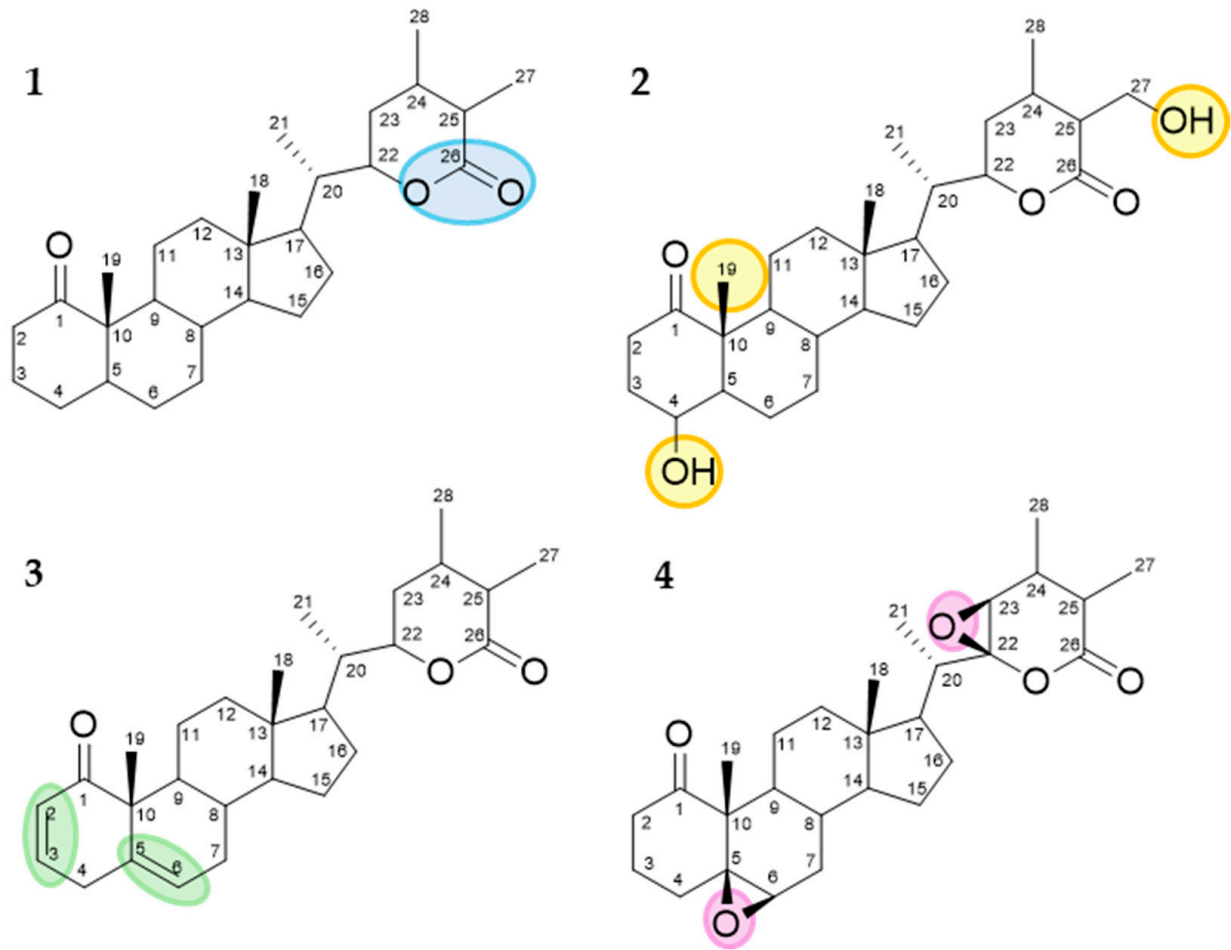
Key structural features associated with increased antitumoral activity. **1**—Lactone ring at C-22 and C-26. **2**—Acylation groups at C-4, C-19, and C-27. **3**—Double bonds at C-1 and C-2 as well as at C-5 and C-6. **4**—Epoxide groups at C-5 and C-6 or C-22 and C-23.

Many studies related to the structure–activity relationship (SAR) for this class of compounds suggest the antitumoral activity of withanolides is closely related to their structural features. Some key structural aspects that contribute to their bioactivity are summarized in Figure 10. The presence of a lactone ring at C-22 and C-26 is pivotal for cytotoxic activity, facilitating interactions with target proteins and cellular membranes. Concerning 2-hydroxyl groups and acylation, the removal of hydroxyl groups at specific positions (C-4, C-19, and C-27) and their acylation can enhance bioavailability. This modification affects the ability of withanolides to interact with cellular targets and induce apoptosis in cancer cells. Furthermore, double bonds within the steroidal nucleus, particularly at C-1 and C-2, as well as C-5 and C-6, are correlated with increased antitumoral activity due to enhanced oxidative stress induction in tumor cells. Additionally, epoxide rings at positions like C-5 and C-6 or C-22 and C-23 contribute to the molecule’s reactivity, facilitating the formation of covalent bonds with biological molecules and thereby disrupting cancer cell proliferation and survival [37,38]. 

The importance of α, β-unsaturated ketone in ring A, 5,6-epoxy group, and a nine-carbon side chain substituted at C-17, featuring an α, β-unsaturated-δ-lactone is considered crucial to the cytotoxicity of this compound class [37,38]. All five known compounds more cytotoxic (**2**, **3**, **7**, **8** and **9**) have the lactone ring and the α, β-unsaturated ketone in ring A, and only physaminilide H (**8**) and aurelianolide B (**9**) lack the epoxide ring. However, despite the absence of the 5,6-epoxy group, the cytotoxicity of physaminilide H (**8**) has already been reported in A375 human melanoma cells [23], and the cytotoxicity of aurelianolide B (**9**) has been reported in leukemic cells [7]. The role of the 5,6-epoxy group can be observed by comparing the in vitro cytotoxicity of aurelianolides A (**7**) and B (**9**) against three leukemia cell lines. The influence of the epoxy group present in aurelianolide A (**7**) on molecule reactivity can be evidenced by the lower IC_50_ values of 2.62 µM, 6.47 µM, and 1.41 µM across the three leukemia cell lines of K562, K562-Lucena 1, and Jurkat, respectively. Comparing with aurelianolide B (**9**), with no epoxy group on C5-C6, showed IC_50_ values of 5.07 µM, 16.23 µM, and 2.25 µM for K562, K562-Lucena 1, and Jurkat cells, respectively. Thus, the presence of epoxy groups doubled the cytotoxic effect [7]. The 16-deoxyphiladelphicalactone C (**2**) molecule showed cytotoxic activity against a panel of MM cells [39]; however, it had no antiproliferative properties against LNCaP, ACHN, UO-31, M14, and SK-MEL-28 cells [18]. Withaneomexolide A (**3**) did not show cytotoxicity against MDAMB-231 and MCF-7 cancer cells [19]. 

Among the other compounds with minor cytotoxic activity, only physagulide (**16**) has all three functional groups highlighted as relevant for the cytotoxicity of the withanolides. Nevertheless, it did not show growth inhibitory activity in three human cancer cell lines: human hepatoma (HepG2), human breast carcinoma (MCF-7), and human osteosarcoma (MG-63) cells [30]. Some compounds such as virginol A (**1**), 20S,22R,24S,25S,26R/S)-15α-acetoxy-5,6β:22,26: diepoxy-24-methoxy-4β,25,26trihydroxyergost-2-en-1-one (**4**), physapubescin H (**6**), capsisteroid G (**18**), and capsisteroid D (**19**) lack an α, β-unsaturated-δ-lactone. Among these compounds, virginol A (**1**) has not yet been tested in tumor cells, while (20S,22R,24S,25S,26R/S)-15α-acetoxy-5,6β:22,26:diepoxy-24-methoxy-4β,25,26trihydroxyergost-2-en-1-one (**4**) has potent growth inhibitory effects against four human renal cell carcinoma (RCC) cell lines: 786-O, A-498, Caki-2, and ACHN [32]. Physapubescin H (**6**) has shown cytotoxicity against prostate cancer cells (C4-2B and 22Rvl), renal carcinoma cells (786-O, A-498, Caki-2, and ACHN), and melanoma cells (A375 and A375-S2) [22]. Capsisteroid G (18) had a moderate antiproliferative effect against erythroleukemia (K562), human promyelocytic leukemia (HL-60), acute lymphoblastic leukemia (Molt-4), and liver and bile duct carcinoma (HuCCT1) cells [31], while capsisteroid D (**19**) had no cytotoxicity against erythro myeloblastoid leukemia (K562), human acute lymphoblastic leukemia (Molt-4), and human promyelocytic leukemia (HL-60) cell lines [32]. (4S,20S,22R)-4-Hydroxy-1-oxo-witha-2,5,16,24-tetraenolide (**5**) has shown a moderate cytotoxic activity contra HeLa, A-549, and MCF-7 [21], while the cytotoxicity of withaperuvin N (**12**) and physanicandrolide B (**15**) have not been studied. Baimantuoluoline R (**13**) has moderate cytotoxicity against SGC-7901 [27], and physaminilide B (**14**) exhibited significant antiproliferative activity against melanoma A375 [28]. The molecules with the most different structures in this group are nicanlode C (**10**) and phenowithanolide (**11**). The first was not cytotoxic to HL-60, SMMC-7721, A-549, MCF-7, and SW480 [24], and phenowithanolide (**11**) did not have cytotoxic activity against human lung carcinoma cells (A549) and human colorectal adenocarcinoma cells (DLD-1) [25].

## 3. Materials and Methods

### 3.1. Plant Material

The species *Athenaea fasciculata* (Vell.) I.M.C. Rodrigues & Stehmann was collected in the city of Simão Pereira (MG) and identified by the botanist Dr. Rita de Cassia Almeida-Lafetá (UFRJ), who deposited an exsiccate in the Herbarium of UFRJ, Rio de Janeiro, Brazil, under number 40829. This study was registered in the National Genetic Heritage and Associated Traditional Knowledge Management System (SisGen) under the number AB5D582.

### 3.2. A. fasciculata Sample Preparation (Extract and Partition)

Fresh leaves of *A. fasciculata* were dried in an oven with air circulating at 40 °C for 48 h. After drying, the material (450.0 g) was reduced to small fragments in a knife mill. Part of the powder material (250 g) was extracted by dynamic maceration at room temperature, first with *n*-hexane for 48 h, followed by ethanol for 48 h. In addition, in a similar procedure, another 250 g of plant material was extracted with *n*-hexane for 48 h, followed by methanol for 48 h. Each extract was filtered and separately concentrated under reduced pressure using a rotary evaporator with a heating bath R-210 Buchi (Flawil, Switzerland) equipped with a V-700 Buchi vacuum pump (Flawil, Switzerland), resulting in 11.35 g (*n*-hexane), 23.15 g (ethanol), and 35.10 g (methanol) crude extracts. The dry ethanolic extract obtained was then resuspended in MeOH/H_2_O (3:7) to obtain the liquid–liquid partitioning in increasing order of polarity with *n*-hexane, dichloromethane, ethyl acetate, and butanol (500 mL each). The solvents were evaporated under reduced pressure, yielding the following partitions: *n*-hexane (4.89 g), AFFD (dichloromethane, 4.10 g), AFFAc (ethyl acetate, 2.90 g), AFFBu (butanol, 6.75 g), and AFFAq (aqueous residue, 2.15 g). All solvents used were of spectroscopic grade and were obtained from Sigma-Aldrich, Darmstadt, Germany.

### 3.3. Cytotoxicity Assays and Statistical Analysis

The cytotoxicities of the extracts and partitions of *A. fasciculata* were assessed at various concentrations ranging from 0.015 µg/mL to 150 µg/mL against K562, K562-Lucena 1, and Jurkat human leukemia cell lines. Human myelogenous K562 (ATCC CCL-243), K562-Lucena 1 (BCRJ 0127; a multidrug-resistant subline of K562), and acute T cell leukemia Jurkat (ATCC TIB182) were kindly provided by Dr Juliana Echevarria-Lima (Paulo Góes Institute of Microbiology; UFRJ, Brazil).

Twenty-four hours before treatment with the samples, 100 μL of the cell suspension (5 × 10^3^ cells/mL) was added to 96-well plates and maintained in a 5% CO_2_ atmosphere at 37 °C. Treatment with the aurelianolides was performed as mentioned above, with each concentration in triplicate. The negative control consisted of cells incubated with vehicle (0.25% of cell culture-grade DMSO in medium; SIGMA), while staurosporine (1 µM) was used as the positive control. After 48 h, 3-[4,5-dimethylthiazol-2-yl]-2,5-diphenyltetrazolium bromide (MTT; SIGMA) was added to the wells and the plates maintained in a CO_2_ incubator for 4 h. Following this incubation period, the plates were centrifuged at 1500 rpm for ten minutes, and the supernatant was aspirated. Formazan crystals, formed by cellular activity, were solubilized with DMSO (SIGMA) [7]. 

Absorbance was measured at 540 nm wavelength, using a microplate reader (Synergy H1M2F; Biotek, Shoreline, WA, USA). The IC_50_ values were determined using GraphPad Prism version 5 (San Diego, CA, USA).

### 3.4. Liquid Chromatography–Mass Spectrometry Analysis

The samples, at a concentration of 2 μg·mL^−1^, underwent analysis utilizing a UHPLC Dionex Ultimate 3000 system (ThermoFischer Scientific, Waltham, MA, USA) connected to an LCQfleet mass spectrometer (ThermoFischer Scientific, Waltham, MA, USA). A Supelcosil LC-18 (5 μm) column was utilized for chromatography. The mobile phase consisted of water with 0.1% formic acid (A) and acetonitrile (B). The flow rate was maintained at 0.30 mL.min^−1^ and the elution gradient programming was as follows: 0–0.6 min (10% B), 0.6–2.8 min (10–40% B), 2.8–3.3 min (40% B), 3.3–5.6 min (40–50% B), 5.6–6.1 min (50% B), 6.1–7.8 min (50–60% B), 7.8–8.3 min (60% B), 8.3–9.2 min (60–80% B), 9.2–10.6 min (80% B), 10.6–12 min (80–10% B), and 12–15.3 min (10% B). 

The mass spectrometer, equipped with an electrospray ionization source (ESI) and an ion trap analyzer, was operated in positive and negative ionization modes. Mass spectra were acquired within the *m*/*z* range of 100–1000. Collision-induced dissociation (CID) was performed with a normalized collision energy of 35 eV, using high-purity helium (He) as the collision gas. MS/MS fragmentation (TANDEM) of the three most intense ions was performed with counts above 100 per scan. Each analysis was conducted in triplicate, resulting in a total of 24 analyses.

### 3.5. Data Processing and Chemometric Analysis

The LC-MS data were converted to the mzML format using the Proteowizard—MSconvert version 3.02 tool. Subsequently, MZmine v. 2.53 software was employed for data processing, including mass detection, mass chromatogram construction, wavelet deconvolution, isotopic removal, and data alignment. The processed chemical data (*m*/*z* ions and retention time), along with the biological data (IC_50_), were exported to UnscramblerX software version 10.4 for analysis. The objective was to predict potential cytotoxic compounds against the three leukemia cell lines. Chemometric analyses were conducted using low-level data fusion (concatenated ESI [+/−] data) and a Partial Least Squares (PLS) regression model. In the PLS regression, samples were categorized into two groups (binary values) based on average IC_50_ values: cytotoxic samples (IC_50_ ≤ 100 μg·mL^−1^—[1]) and non-cytotoxic samples (IC_50_ > 100 μg−mL^−1^—[−1]). The NIPALS algorithm was employed to construct the PLS model, along with the Leave-One-Out cross-validation method.

### 3.6. Molecular Networking and Annotation of Compounds

The data obtained from MZmine were uploaded to the open-source Global Natural Products Social (GNPS) online platform, along with the converted files (.mzML) and metadata table. These files were utilized to construct molecular networks and dereplicate the predicted compounds using a Partial Least Squares (PLS) regression model. In addition to GNPS annotation, a custom database was employed. The analyses adhered to the parameters recommended in the GNPS documentation for unity resolution data. The data generated by GNPS were then downloaded and processed in Cytoscape 3.9.1 to extract the results of the molecular networking analysis.

## 4. Conclusions

Currently, a substantial number of withanolides have been isolated and characterized. These compounds have attracted significant attention due to their promising potential as antitumoral agents, owing to their ability to target multiple pathways related to their complex structural features. Therefore, understanding the SAR of withanolides is essential for the development of new and more effective anticancer therapies, as well as for the creation of novel analogs with enhanced efficacy and reduced toxicity. In this study, UHPLC-MS/MS, in combination with bioactivity-based molecular networking (GNPS), provided a comprehensive and rapid methodology for the identification of 22 known withanolides derived from *Athenaea fasciculata*. This approach facilitated the correlation of ions associated with the most cytotoxic compounds, present in the dichloromethane partition of the ethanolic extract of *A. fasciculata*, across various leukemia cell lines such as Jurkat, K562, and K562-Lucena 1. Furthermore, an integrated, practical, and targeted data processing strategy was employed alongside an in-house database, enabling the rapid profiling of these constituents for the first time. The withanolides identified in this study warrant further isolation for in vitro or in vivo activity screening. Ongoing research into *A. fasciculata* continues to reveal its potential as a powerful source of new natural alternative compounds in the fight against cancer and holds promise for future drug research and development.

## Figures and Tables

**Table 1:** In vitro cytotoxicity of *A. fasciculata* extracts and partitions against leukemia cell lines.

Material	IC_50_ (µg/mL)		
	Jurkat	K562	K562-Lucena 1
Methanolic extract (AFFM)	67.70	108.00	255.20
Hexanic extract (AFFH)	50.08	104.30	84.81
Ethanolic extract (AFFE)	55.21	98.88	110.80
Ethanolic extract hexane fraction (AFFPH)	55.29	92.31	97.13
Ethanolic extract dichloromethane fraction (AFFD)	14.34	26.50	38.64
Ethanolic extract ethyl acetate fraction (AFFAc)	92.21	384.70	>1000
Ethanolic extract butanol fraction (AFFBu)	418.4	716.5	>1000
Ethanolic extract aqueous residue (AFFAq)	>1000	>1000	>1000

## Data Availability

Data are contained within the article and Supplementary Materials.

## Supplementary Materials

The following supporting information can be downloaded at: https://www.mdpi.com/article/10.3390/molecules29184357/s1, Figure S1. MS/MS spectrum of compound **1** at *m*/*z* 528.9 [M − H]^−^ (virginol A). Figure S2. MS/MS spectrum of compound **2** at *m*/*z* 486.9 [M − H]^−^ (16-deoxyphiladelphicalactone C). Figure S3. MS/MS spectrum of compound **3** at *m*/*z* 542.9 [M − H]^−^ (withaneomexolide A). Figure S4. MS/MS spectrum of compound **4** at *m*/*z* 560.9 [M − H]^−^ (20S,22R,24S,25S,26R/S)-15α-acetoxy-5,6β:22,26:diepoxy-24-methoxy-4β,25,26-trihydroxyergost-2-en-1-one). Figure S5. MS/MS spectrum of compound **5** at *m*/*z* 434.9 [M − H]^−^ ((4S,20S,22R)-4-Hydroxy-1-oxo-witha-2,5,16,24-tetraenolide). Figure S6. MS/MS spectrum of compound **6** at *m*/*z* 512.8 [M − H]^−^ (physapubescin H). Figure S7. MS/MS spectrum of compound **7** at *m*/*z* 526.9 [M − H]^−^ (aurelianolide A). Figure S8. MS/MS spectrum of compound **8** at *m*/*z* 508.9 [M − H]^−^ (physaminilide H). Figure S9. MS/MS spectrum of compound **8** at *m*/*z* 510.9 [M + H]^+^ (physaminilide H). Figure S10. MS/MS spectrum of compound **9** at *m*/*z* 510.9 [M − H]^−^ (aurelianolide B). Figure S11. MS/MS spectrum of compound **9** at *m*/*z* 512.9 [M + H]^+^ (aurelianolide B). Figure S12. MS/MS spectrum of compound **10** at *m*/*z* 478.9 [M − H]^−^ (nicanlode C). Figure S13. MS/MS spectrum of compound **11** at *m*/*z* 419.0 [M − H]^−^ (phenowithanolide). Figure S14. MS/MS spectrum of compound **12** at *m*/*z* 519.2 [M − H]^−^ (withaperuvin N). Figure S15. MS/MS spectrum of compound **13** at *m*/*z* 496.9 [M − H]^−^ (baimantuoluoline). Figure S16. MS/MS spectrum of compound **14** at *m*/*z* 526.9 [M + H]^+^ (physaminilide B). Figure S17. MS/MS spectrum of compound **15** at *m*/*z* 560.9 [M + H]^+^ (physanicandrolide B). Figure S18. MS/MS spectrum of compound **16** at *m*/*z* 542.9 [M + H]^+^ (physagulide C). Figure S19. MS/MS spectrum of compound **17** at *m*/*z* 492.9 [M + H]^+^ (capsisteroid G). Figure S20. MS/MS spectrum of compound **18** at *m*/*z* 508.9 [M + H]^+^ (capsisteroid D). Figure S21. MS/MS spectrum of compound **19** at *m*/*z* 519.2 [M + H]^+^ (withalongolide D).
