# Supplementary material for: An Integrated Strategy of UHPLC-ESI-MS/MS Combined with Bioactivity-Based Molecular Networking for Identification of Antitumoral Withanolides from Athenaea fasciculata (Vell.) I.M.C. Rodrigues & Stehmann
Source: Molecules. 2024 Sep 13;29(18):4357. doi: 10.3390/molecules29184357 (PMC11434275; doi:10.3390/molecules29184357)

Supplementary material

An integrated strategy of UHPLC-ESI-MS/MS combined with bioactivity-based molecular networking for identification of antitumoral withanolides from *Athenaea fasciculata* (Vell.) I.M.C. Rodrigues & Stehmann

André Mesquita Marques [1], Lavinia de Carvalho Brito [1,*], Simony Carvalho Mendonça [2], Brendo Araujo Gomes [2], Flávia da Cunha Camillo [1], Gustavo Werneck de Souza e Silva [3], André Luiz Franco Sampaio [3], Suzana Guimarães Leitão [2] and Maria Raquel Figueiredo [1]

1 Department of Natural Products, Pharmaceutical Technology Institute, Farmanguinhos, Fiocruz, Sizenando Nabuco 100 st, Manguinhos, Rio de Janeiro 21041-250, Brazil; andrefarmaciarj@yahoo.com.br (A.M.M.); flavia.camillo@fiocruz.br (F.C.C.); maria.figueiredo@fiocruz.br (M.R.F.)

2 Department of Natural Products and Food, Faculty of Pharmacy, Center of Health Sciences (CCS), Federal University of Rio de Janeiro, Rio de Janeiro 21941-902, Brazil; sy2802@gmail.com (S.C.M.); brendoo.bc@gmail.com (B.A.G.); sgleitao@gmail.com (S.G.L.)

3 Laboratory of Molecular Pharmacology, Pharmaceutical Technology Institute, Farmanguinhos, Fiocruz, Avenida Brasil 4365, Manguinhos, Rio de Janeiro 21041-250, Brazil; gustavo.werneck@fiocruz.br (G.W.S.S.); andre.sampaio@fiocruz.br (A.L.F.S.)

* Correspondence: laviniabrito@yahoo.com.br

Figure S1. MS/MS spectrum of compound **1** at *m/z* 528.9 $[M\text{-}H]^-$ (virginol A)

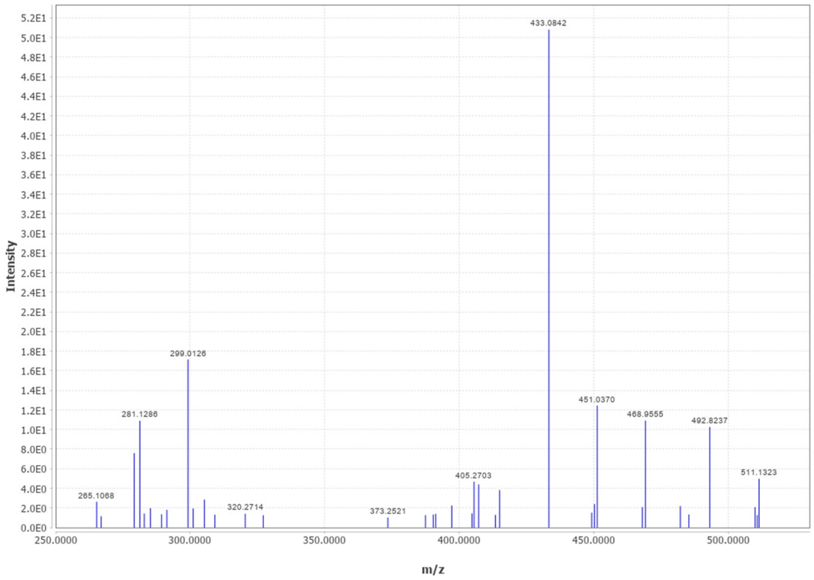

Figure S2. MS/MS spectrum of compound **2** at *m/z* 486.9 $[M\text{-}H]^-$ (16-deoxyphiladelphicalactone C)

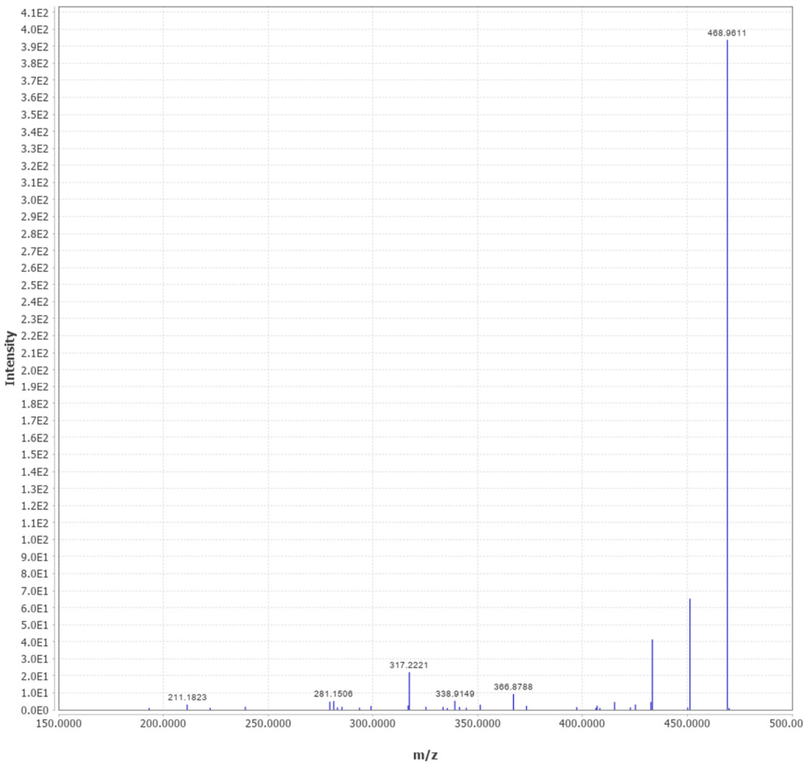

Figure S3. MS/MS spectrum of compound **3** at *m*/*z* 542.9 [M-H]$^{-}$ (withaneomexolide A).

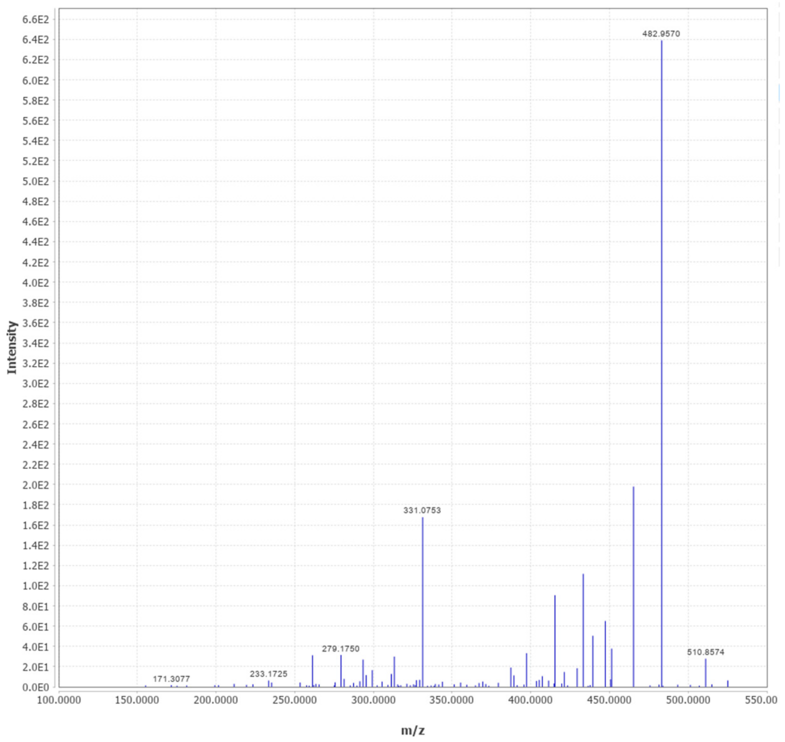

Figure S4. MS/MS spectrum of compound **4** at *m/z* 560.9 [M-H]⁻ (20S,22R,24S,25S,26R/S)-15α-acetoxy-5,6β:22,26:diepoxy-24-methoxy-4β,25,26-trihydroxyergost-2-en-1-one).

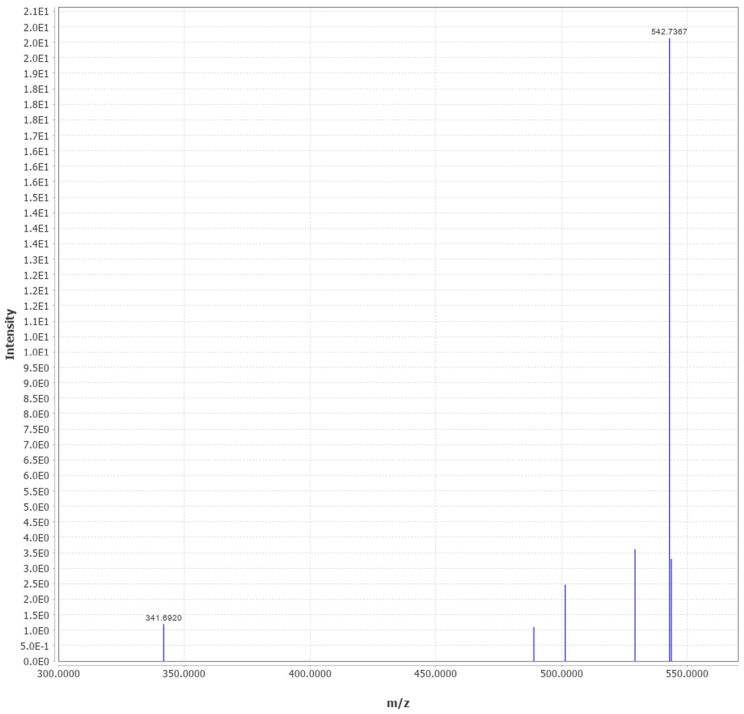

Figure S5. MS/MS spectrum of compound **5** at *m/z* 434.9 [M-H]⁻ ((4S,20S,22R)-4-Hydroxy-1-oxo-witha-2,5,16,24-tetraenolide).

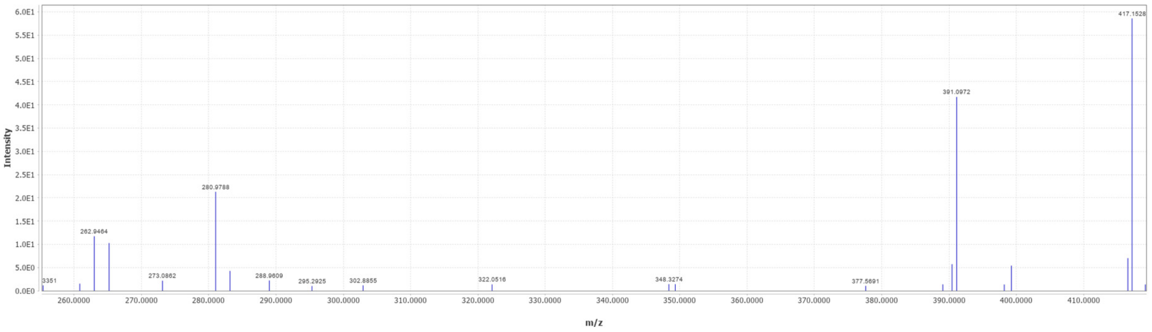

Figure S6. MS/MS spectrum of compound **6** at *m/z* 512.8 [M-H]⁻ (physapubescin H).

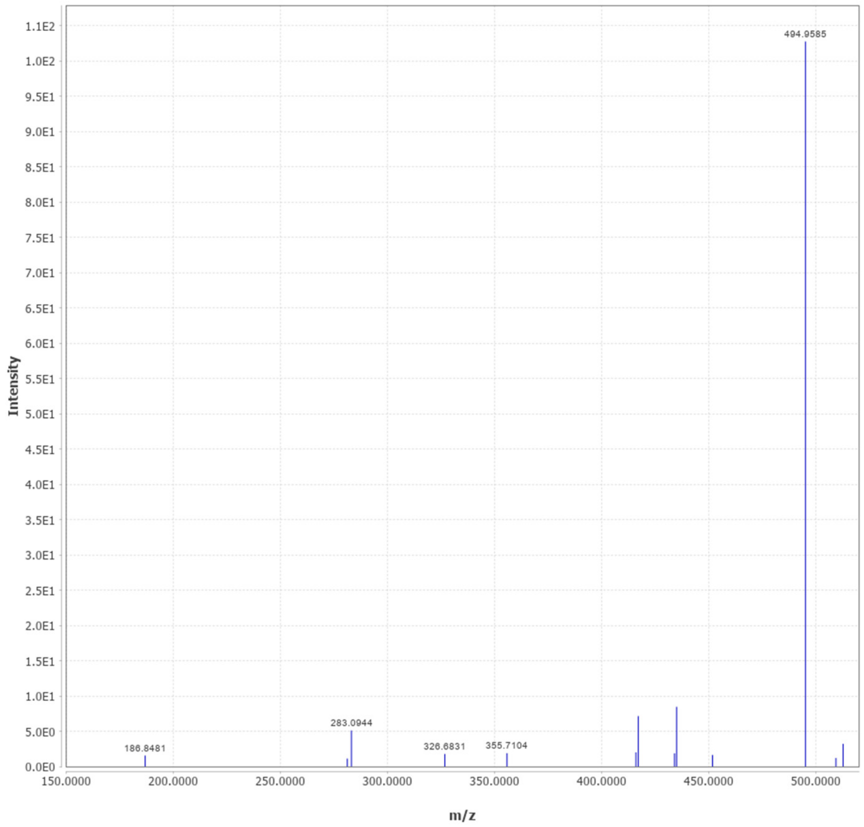

Intensity
m/z
494.9585
283.0944
186.8481
326.6831
355.7104

Figure S7. MS/MS spectrum of compound **7** at *m/z* 526.9 [M-H]$^-$ (aurelianolide A).

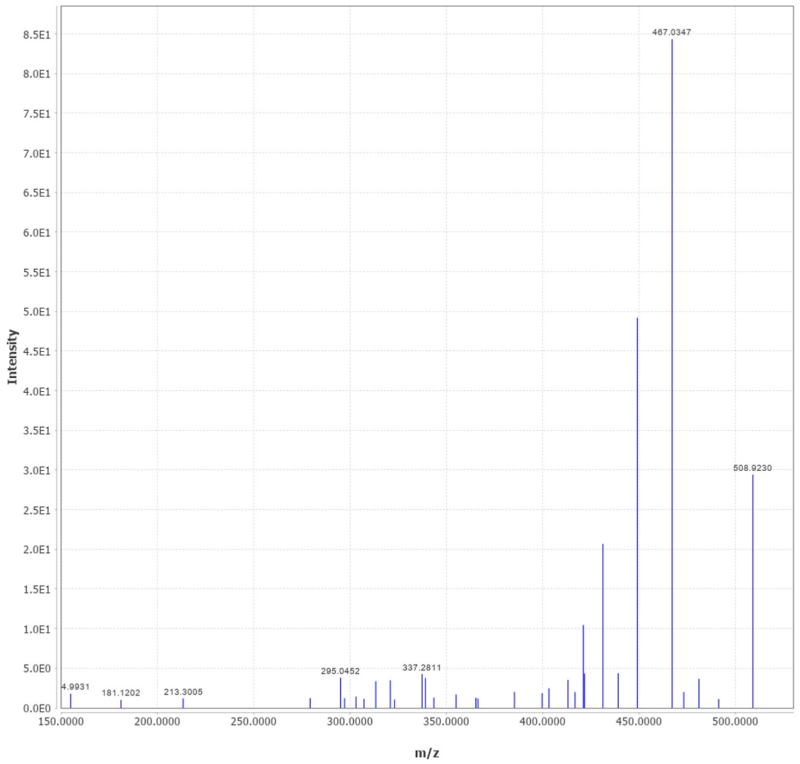

Figure S8. MS/MS spectrum of compound **8** at *m/z* 508.9 [M-H]$^-$ (physaminilide H).

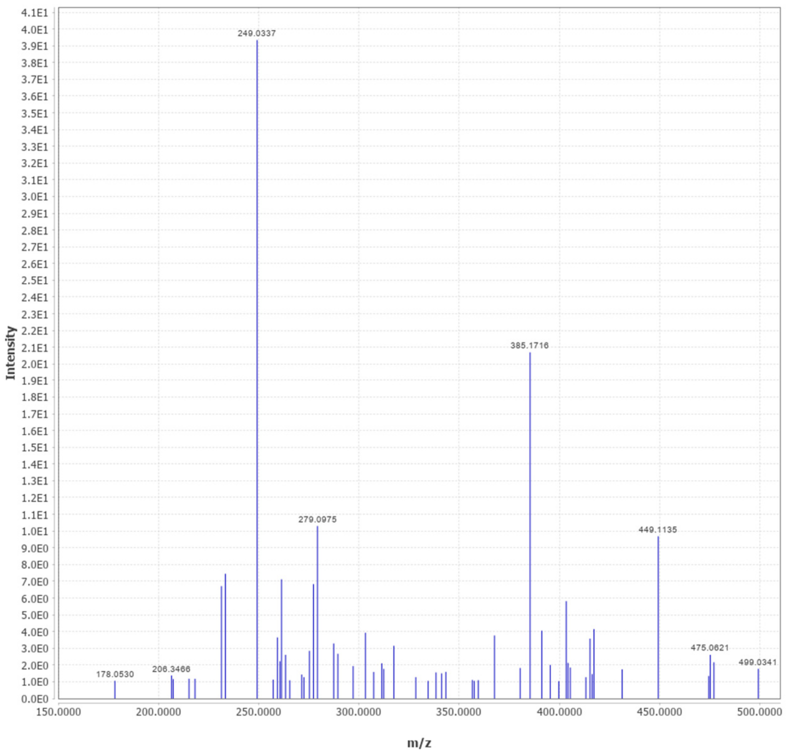

Figure S9. MS/MS spectrum of compound **8** at *m/z* 510.9 $[M+H]^+$ (physaminilide H).

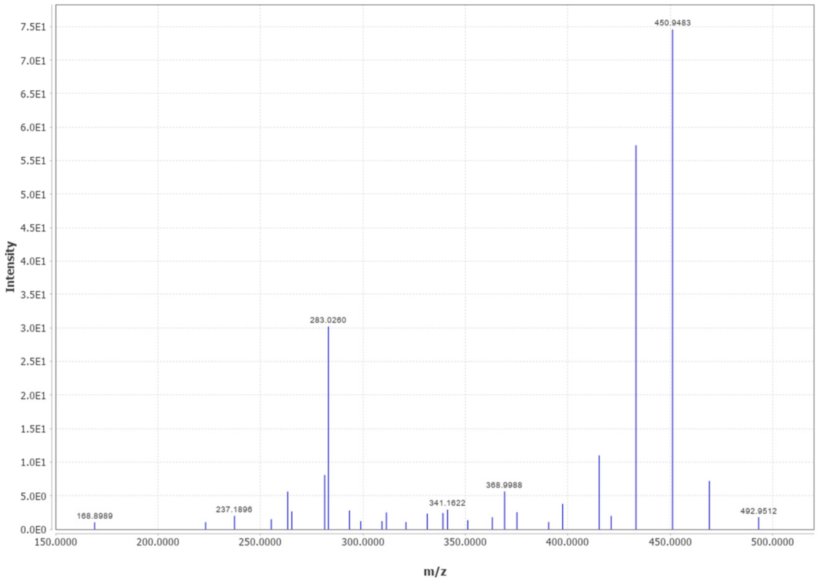

Figure S10. MS/MS spectrum of compound **9** at *m/z* 510.9 $[M-H]^-$ (aurelianolide B).

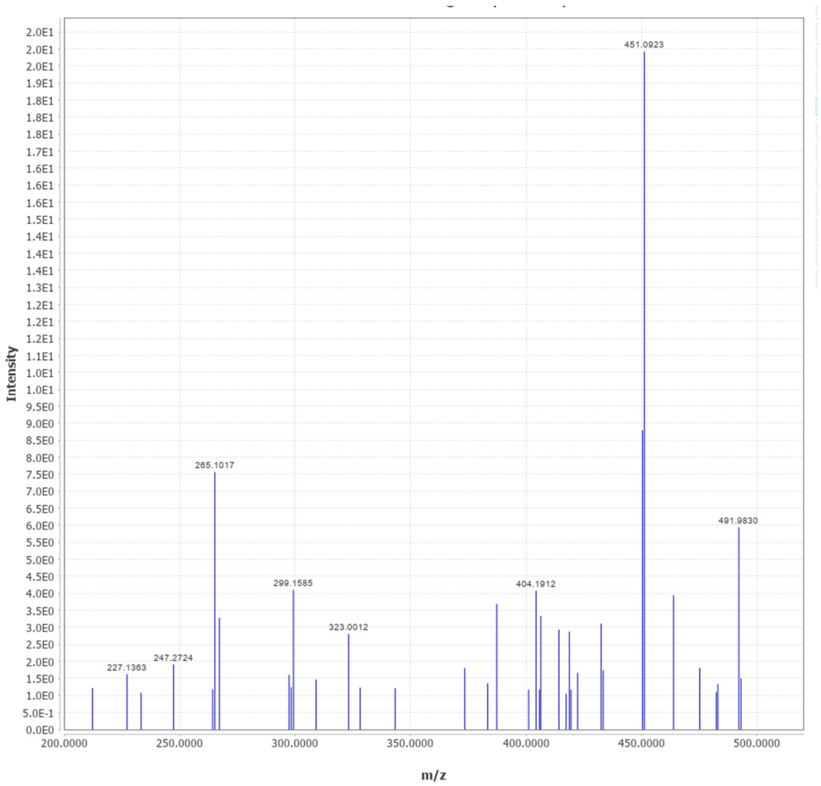

Figure S11. MS/MS spectrum of compound **9** at *m*/*z* 512.9 [M+H]+ (aurelianolide B).

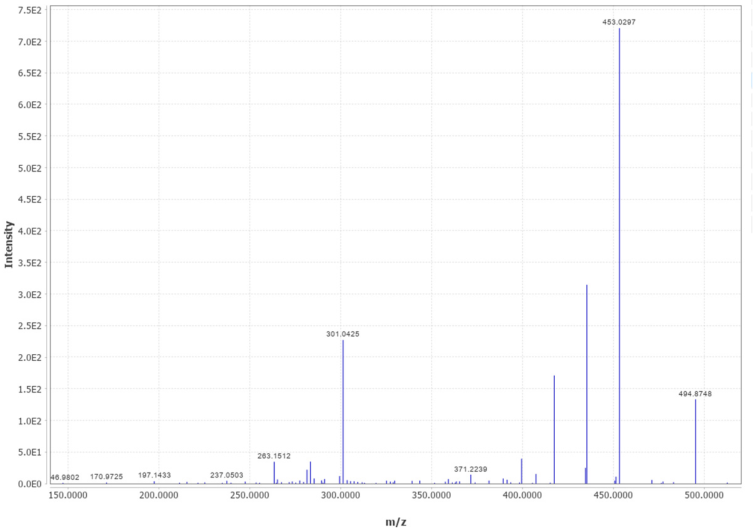

Figure S12. MS/MS spectrum of compound **10** at *m*/*z* 478.9 [M-H]- (nicanlode C).

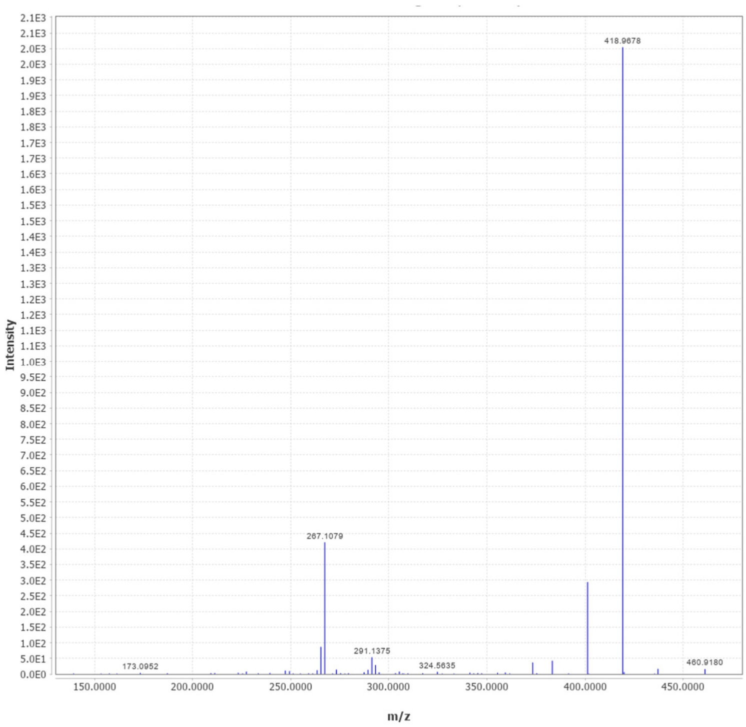

Figure S13. MS/MS spectrum of compound **11** at *m/z* 419.0 [M-H]$^-$ (phenowithanolide).

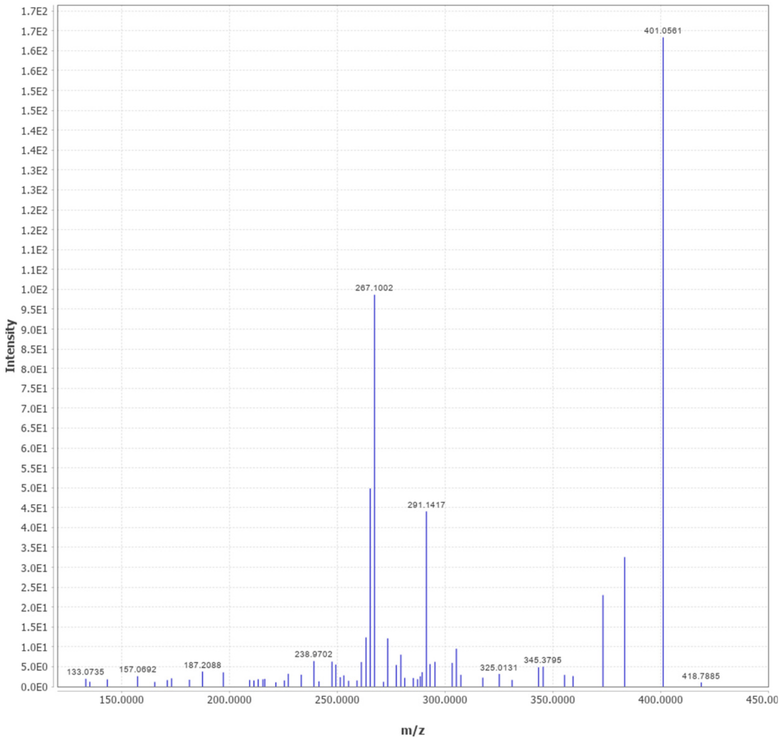

Figure S14. MS/MS spectrum of compound **12** at *m/z* 519.2 [M-H]$^-$ (withaperuvin N).

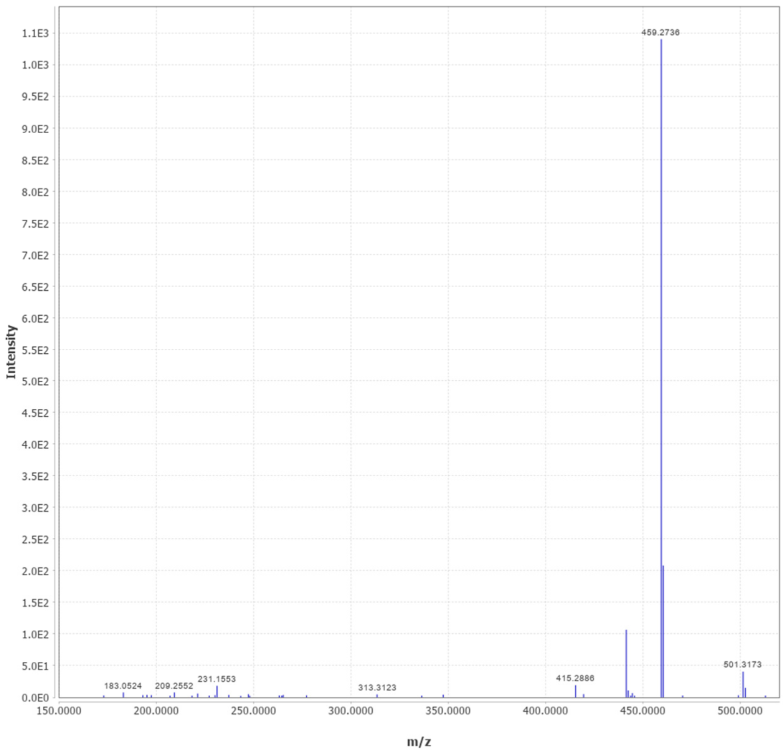

Figure S15. MS/MS spectrum of compound **13** at *m*/*z* 496.9 [M-H]$^-$ (baimantuoluoline).

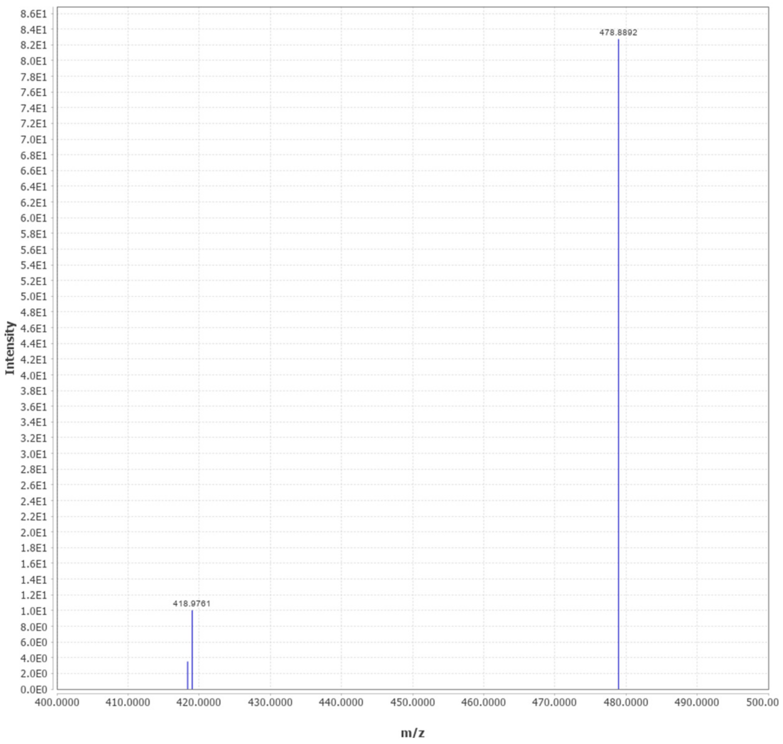

Figure S16. MS/MS spectrum of compound **14** at *m*/*z* 526.9 [M+H]$^+$ (physaminilide B).

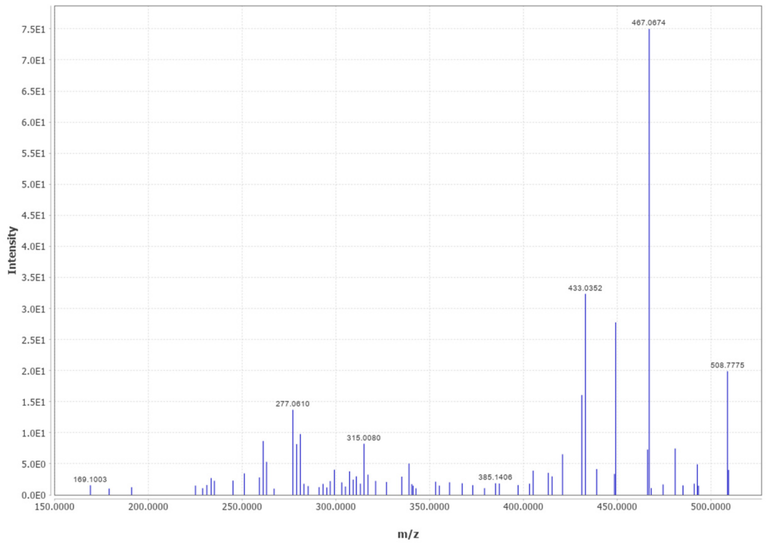

Figure S17. MS/MS spectrum of compound **15** at *m*/*z* 560.9 $[M+H]^+$ (physanicandrolide B).

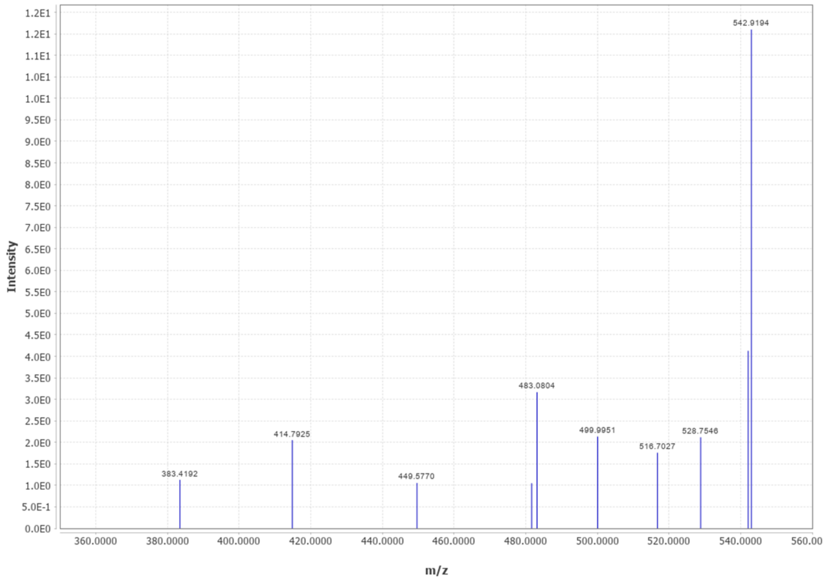

Figure S18. MS/MS spectrum of compound **16** at *m*/*z* 542.9 $[M+H]^+$ (physagulide C).

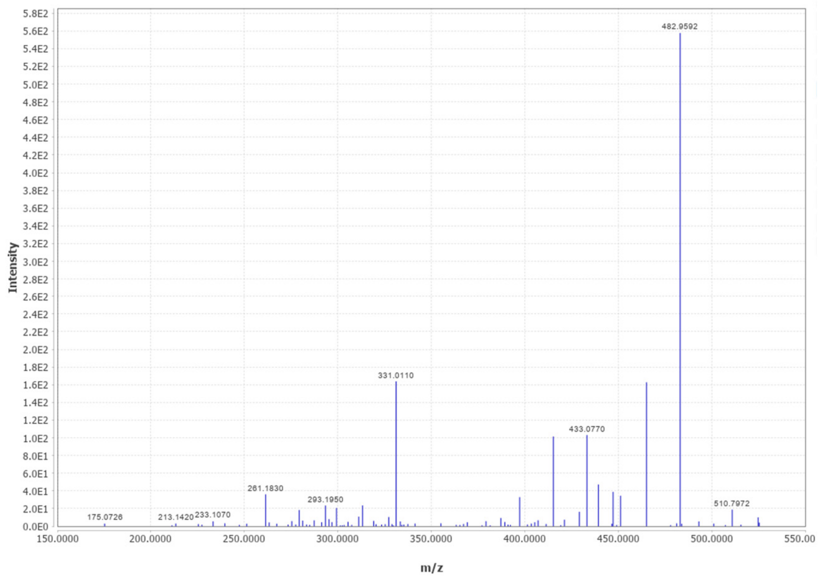

Figure S19. MS/MS spectrum of compound **17** at *m/z* 492.9 $[M+H]^+$ (capsisteroid G).

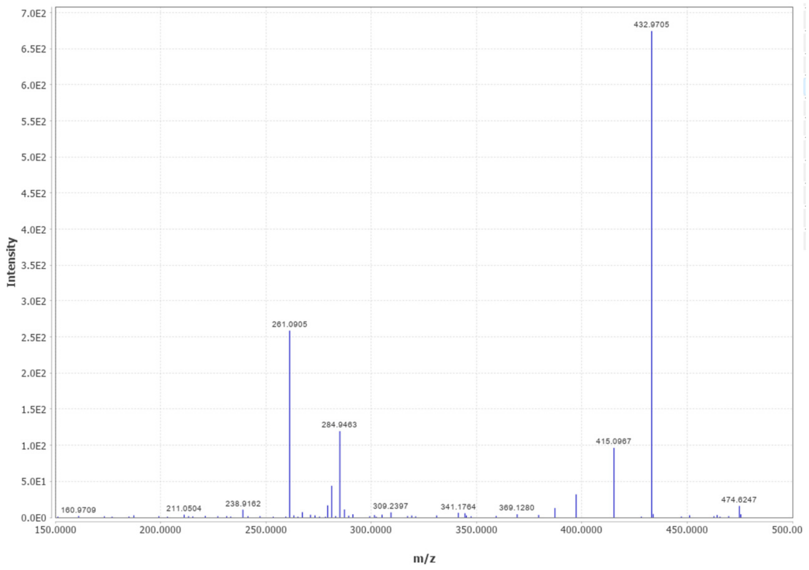

Figure S20. MS/MS spectrum of compound **18** at *m/z* 508.9 $[M+H]^+$ (capsisteroid D).

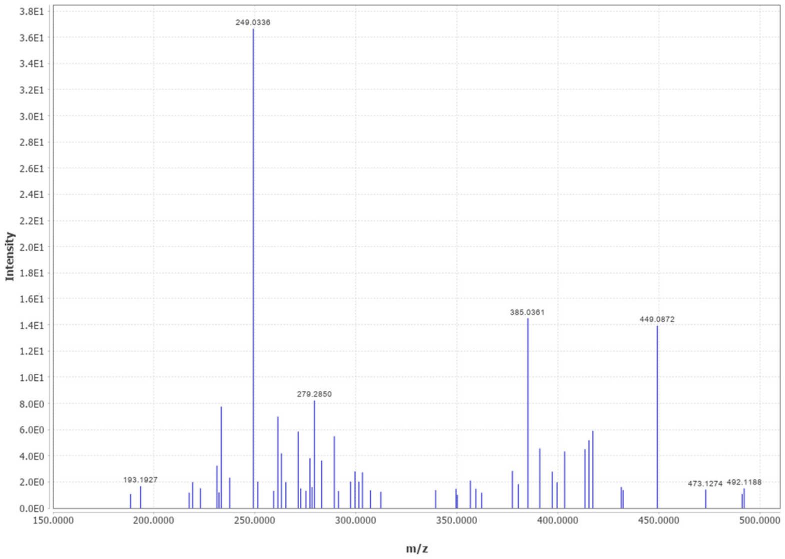

Figure S21. MS/MS spectrum of compound **19** at *m*/*z* 519.2 $[M+H]^+$ (withalongolide D).

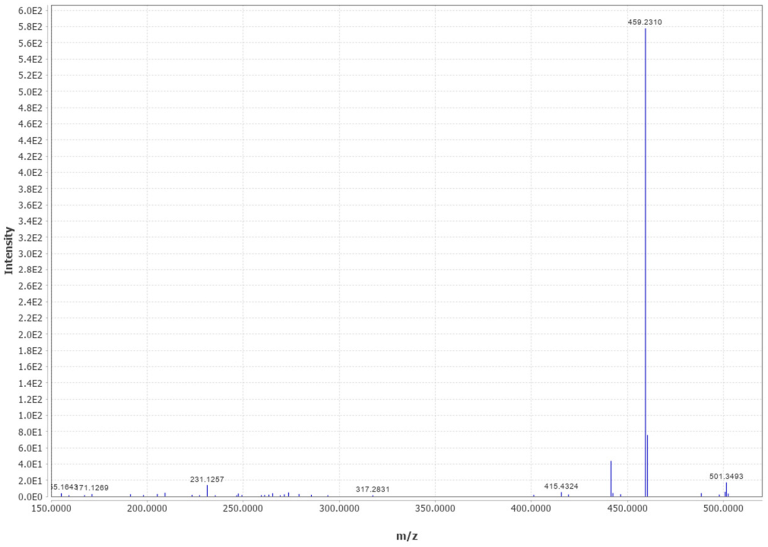

Supplement: Supplementary file 1 [file molecules-29-04357-s001.zip › molecules-3141708-supplementary.pdf]
